# Supplementary material for: Longitudinal Morphological Changes in the Adenoids and Tonsils in Japanese School Children
Source: J Clin Med. 2021 Oct 26;10(21):4956. doi: 10.3390/jcm10214956 (PMC8584668; doi:10.3390/jcm10214956)
Supplement: Supplementary file 1 [file jcm-10-04956-s001.zip › jcm-1404791-supplementary.pdf]

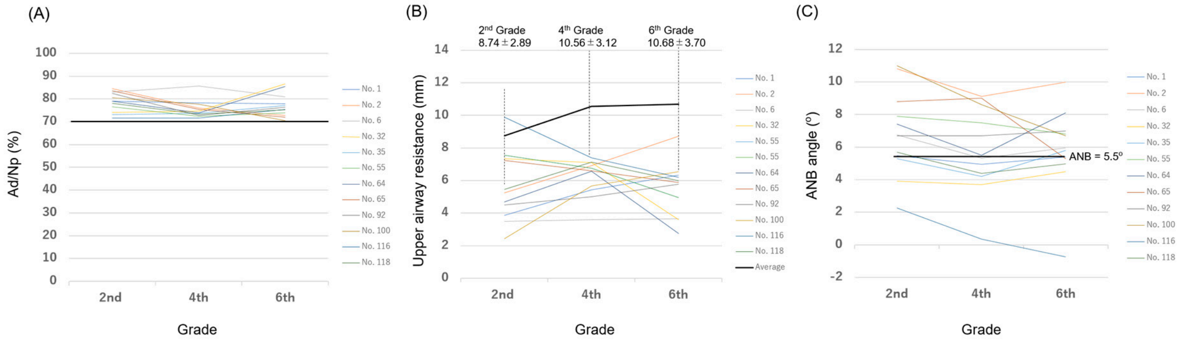

Supplemental Figure S1. Changes in adenoid/nasopharynx (Ad/Np) and upper airway resistance (UAR) with grade of 12 school children who showed more than 70% of the Ad/Np through the study period. (A) Grade-dependent change in the Ad/Np. (B) Grade-dependent change in the upper airway resistance. (C) Grade-dependent change in the ANB angle.

Among 99 school children, 12 children showed more than 70% of the adenoid/nasopharynx (Ad/Np) ratio through the experimental period (A). Eleven out of these 12 children also showed less than 7.0 mm of the UAR even at the 6th grade, while the average of the upper airway resistance (UAR) at the 6th grade was 10.7 ± 3.6 mm (B). Furthermore, 7 out of 12 children exhibited more than 5.5 degrees of the ANB angle, indicating they were defined as skeletal Class II jaw-base relationship (C).
